# Supplementary material for: Beneficial Effects of White Grape Pomace in Experimental Dexamethasone-Induced Hypertension
Source: Diseases. 2025 Apr 24;13(5):132. doi: 10.3390/diseases13050132 (PMC12110589; doi:10.3390/diseases13050132)
Supplement: Supplementary file 1 [file diseases-13-00132-s001.zip › diseases-3556135-supplementary.pdf]

diseases

MDPI

Article

Beneficial Effects of White Grape Pomace in Experimental Dexamethasone-Induced Hypertension

Raluca Maria Pop [1], Paul-Mihai Boarescu [2,3,*], Corina Ioana Bocsan [1], Mădălina Luciana Gherman [4], Veronica Sanda Chedea [5], Elena-Mihaela Jianu [6], Ștefan Horia Roșian [7,8], Ioana Boarescu [2,3], Floricuța Ranga [9], Maria Doinița Muntean [5], Maria Comșa [5], Sebastian Armean [1], Ana Uifălean [10], Alina Elena Pârvu [10] and Anca Dana Buzoianu [1]

Table S1. Median values of Systolic Blood Pressure (SBP), Diastolic Blood Pressure (DBP) and Mean Arterial Pressure (MAP) in time.

		SBP			
**Groups**	**SBP_0**	**SBP_4**	**SBP_8**	**SBP_12**	**SBP_16**
CTRL_S	130 (124-133)	130 (125-132)	130 (125-132)	132 (129-135)	130 (127-131)
CTRL_DEXA	131 (138-134)	136 (133-141)	149 (143-151)	153.50 (149-159)	166 (161-168)
DEXA_GP1	130.50 (129-137)	132 (129-133)	139 (134-142)	148 (145-154)	151.5 (148-155)
DEXA_GP2	130 (122-132)	133.50 (130-138)	141 (139-148)	155 (152-158)	156.5 (154-165)
DEXA_LIS	132 (128-134)	129 (122-132)	132 (128-134)	133.50 (131-134)	135 (132-136)
		**TAD**			
**Groups**	**DBP_0**	**DBP_4**	**DBP_8**	**DBP_12**	**DBP_16**
CTRL_S	73 (65-80)	74 (61-80)	70 (65-75)	77 (174-79)	76 (71-82)
CTRL_DEXA	70.5 (68-72)	76.5 (72-80)	92 (90-98)	98 (93-100)	101.5 (96-106)
DEXA_GP1	69 (65-75)	76 (69-81)	83.5 (82-89)	94 (88-98)	93.5 (90-99)
DEXA_GP2	72 (69-75)	78.5 (70-80)	94 (92-95)	98 (88-101)	100.5 (99-103)
DEXA_LIS	72.5 (65-82)	74.50 (65-80)	82 (78-88)	81 (77-85)	80.5 (78-86)
		**MAP**			
**Group**	**MAP_0**	**MAP_4**	**MAP_8**	**MAP_12**	**MAP_16**
CTRL_S	90.83 (88-97.33)	90.33 (83.33-97.67)	88.33 (87-93.33)	95.67 (92.33-97)	93.67 (90-97.67)
CTRL_DEXA	88.83 (88.33-92)	95.33 (93.67-98.33)	110.83 (108-115)	115 (111.67-118.33)	122.50 (119.67-127)
DEXA_GP1	89.50 (86.33-94)	93.50 (89.33-100.33)	103.17 (100-105.67)	110 (108-114.33)	113.17 (111.67-114.67)
DEXA_GP2	91 (88.66-93.66)	96.33 (90.67-98)	109.50 (107.33-111)	115.50 (109.33-120.67)	119.67 (117.33-121.67)
DEXA_LIS	92.66 (84.66-99)	90.67 (86.67-96)	99.33 (94.67-101.67)	99.33 (95-101.33)	99.83 (97-102)

* Values are presented as median (25–75 percentiles).

Table S2. In vivo antioxidant activity of the white grape pomace.

Groups	TAC (mmol Trolox eq/L)	TOS (mmol mmol/L)/eq/L)	NO (mmol/L)	MDA (nmol/L)	Total Thiols (mmol/L)	OSI
CTRL_S	1.096[b,e] (1.093-1.100)	7.72[e] (6.74-8.57)	39.57[b,d,e] (38.76-43.62)	3.44 (3.27-3.61)	368[b,e] (267-587)	7.03[e] (6.16-7.79)
CTRL_DEXA	1.090 (1.090-1.091)	12.09[e] (6.61-14.82)	30.37 (28.31-39.65)	4.39[a,c,e] (3.69-4.49)	252 (233-313)	11.08[e] (6.07-13.58)
DEXA_GP1	1.095[b,e] (1.094-1.096)	9.09[e] (6.87-13.52)	36.04[d,e] (33.90-40.24)	3.61 (3.35-3.82)	420[b,e] (397-471)	8.28[e] (6.28-12.34)
DEXA_GP2	1.095[b,e] (1.094-1.096)	14.17[a,c,e] (13.13-15.35)	31.77 (26.98-33.61)	4.36[a,c,e] (4.18-4.68)	414[b,e] (401-439)	12.94[a,c,e] (12.03-14.03)
DEXA_LIS	1.089 (1.088-1.090)	4.98 (4.13-5.96)	30.07 (25.51-30.96)	3.60 (3.40-3.82)	262 (253-289)	4.57 (3.80-5.43)

Rats were grouped into control groups: CTRL_S – treated with saline, and CTRL_DEXA – treated with dexamethasone, and hypertension groups as follows: DEXA_GP1 treated with white grape pomace concentration 1, DEXA_GP2 treated white grape pomace concentration 2 and DEXA_LIS- treated with lisinopril, where [a] had $p < 0.05$, versus the CTRL_S group; [b] had $p < 0.05$, versus the CTRL_DEXA; [c] had $p < 0.05$, versus the DEXA_WGP1; [d] had $p < 0.05$, versus the DEXA_GP2, and [e] had $p < 0.05$, versus the DEXA_LIS group following Kruskal–WallisKruskal-Wallis Test.
